# 3,9-Bis(2,4-dichloro­phen­yl)-2,4,8,10-tetra­oxaspiro­[5.5]undeca­ne

**DOI:** 10.1107/S1600536810024712

**Published:** 2010-06-30

**Authors:** Zhengyi Li, Qiuzheng Tang, Kang Wu, Shuling Yu, Xiaoqiang Sun

**Affiliations:** a Key Laboratory of Fine Chemical Engineering, Changzhou University, Changzhou 213164, Jiangsu, People’s Republic of China

## Abstract

In the title compound, C_19_H_16_Cl_4_O_4_, the two halves of the mol­ecule are related by a crystallographic twofold rotation axis passing through the central spiro-C atom. The two non-planar six-membered heterocycles both adopt chair conformations, and the dihedral angle between the two benzene rings is 76.6 (1)°. In the crystal structure, inter­molecular C—H⋯O hydrogen bonds link the mol­ecules into chains along the *c* axis.

## Related literature

For general background to spiranes, see: Cismaş *et al.* (2005 ▶); Mihiş *et al.* (2008 ▶); Sun *et al.* (2010 ▶).
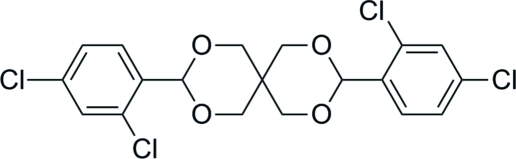


## Experimental

### 

#### Crystal data

C_19_H_16_Cl_4_O_4_
*M* *_r_* = 450.12
Monoclinic, 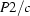

*a* = 14.365 (2) Å
*b* = 5.7397 (9) Å
*c* = 11.7464 (19) Å
β = 93.275 (3)°
*V* = 966.9 (3) Å^3^
*Z* = 2
Mo *K*α radiation
μ = 0.64 mm^−1^
*T* = 295 K
0.21 × 0.21 × 0.16 mm

#### Data collection

Bruker APEXII CCD diffractometer
Absorption correction: multi-scan (*SADABS*; Bruker, 2000 ▶) *T*_min_ = 0.878, *T*_max_ = 0.905
5044 measured reflections
1686 independent reflections
1444 reflections with *I* > 2σ(*I*)
*R*_int_ = 0.022

#### Refinement

*R*[*F* ^2^ > 2σ(*F* ^2^)] = 0.035
*wR*(*F* ^2^) = 0.160
*S* = 1.02
1686 reflections
123 parameters
H-atom parameters constrained
Δρ_max_ = 0.35 e Å^−3^
Δρ_min_ = −0.40 e Å^−3^

### 

Data collection: *SMART* (Bruker, 2000 ▶); cell refinement: *SAINT* (Bruker, 2000 ▶); data reduction: *SAINT*; program(s) used to solve structure: *SHELXTL* (Sheldrick, 2008 ▶); program(s) used to refine structure: *SHELXTL*; molecular graphics: *SHELXTL* (Sheldrick, 2008 ▶); software used to prepare material for publication: *SHELXTL*.

## Supplementary Material

Crystal structure: contains datablocks I, global. DOI: 10.1107/S1600536810024712/ez2218sup1.cif

Structure factors: contains datablocks I. DOI: 10.1107/S1600536810024712/ez2218Isup2.hkl

Additional supplementary materials:  crystallographic information; 3D view; checkCIF report

## Figures and Tables

**Table 1:** Hydrogen-bond geometry (Å, °)

*D*—H⋯*A*	*D*—H	H⋯*A*	*D*⋯*A*	*D*—H⋯*A*
C5—H5⋯O2^i^	0.93	2.58	3.425 (3)	152

Symmetry code: (i) 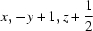.

**Fig. 1.**
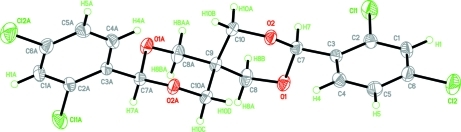
The molecular structure of the title compound showing the atom numbering scheme. Displacement ellipsoids are drawn at the 30% probability level [Symmetry code: -x + 1, y, -z + 1/2].

**Fig. 2.**
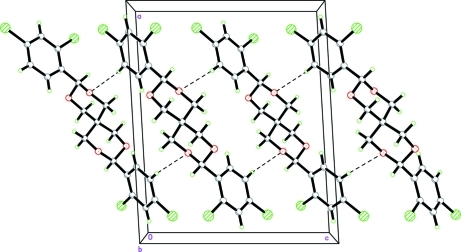
One-dimensional stack running along the c axis. Hydrogen bonds are shown as dashed lines.

## supplementary crystallographic information

### Comment

Owing to their characteristic axial and helical chirality, the stereochemistry of spiranes with six-membered rings has been extensively studied (Cismaş *et al.*, 2005). In the past three decades, most of these investigations were carried out with spiranes containing 1,3-dioxane units (Mihiş *et al.*, 2008; Sun *et al.*, 2010). We herein present the structure of 3,9-bis(2,4-dichlorophenyl)-2,4,8,10-tetraoxaspiro[5.5]undecane (Fig. 1).

In the title compound, a 2-fold rotation axis passes through the central spiro-C atom (C9). The two non-planar six-membered heterocycles [(O1, O2 and C7–C10) and (O1A, O2A and C7A–C10A)] both adopt chair conformations, and the dihedral angle between the two benzene rings (C1–C6 and C1A–C6A) is 76.6 (1)°. In the crystal structure, intermolecular C—H···O hydrogen bonds link the molecules to form one-dimensional chain along the *c* axis (Fig. 2).

### Experimental

To a solution of 2,4-dichlorobenzaldehyde (5 mmol, 0.88 g) and pentaerythritol (3 mmol, 0.41 g) in toluene (25 ml), phosphotungstic acid (1 mol%, 16.5 mg) was added as catalyst. The mixture was refluxed for 6 h to complete the reaction. After reaction, the mixture was allowed to cool to room temperature, and dichloromethane (25 ml) was added to dissolve the product. The insoluble residues were filtered off and the filtrate was dried over anhydrous Na_2_SO_4_. The solvent was evaporated under vacuum and the product recrystallized from ethanol to afford a white solid (71% yield, m.p. 469–470 K). Single crystals suitable for X-ray diffraction were also obtained by evaporation of an ethanol solution.

### Refinement

All H atoms were placed in geometrically idealized positions and constrained to ride on their parent atoms, with C—H distances of 0.93–0.98 Å, and with *U*_iso_(H) = 1.2*U*_eq_(C).

### Crystal data

**Table:**

C_19_H_16_Cl_4_O_4_	*F*(000) = 460
*M**_r_* = 450.12	*D*_x_ = 1.546 Mg m^−^^3^
Monoclinic, *P*2/*c*	Mo *K*α radiation, λ = 0.71073 Å
*a* = 14.365 (2) Å	Cell parameters from 2876 reflections
*b* = 5.7397 (9) Å	θ = 2.8–29.5°
*c* = 11.7464 (19) Å	µ = 0.64 mm^−^^1^
β = 93.275 (3)°	*T* = 295 K
*V* = 966.9 (3) Å^3^	Block, colorless
*Z* = 2	0.21 × 0.21 × 0.16 mm

### Data collection

**Table:**

Bruker APEXII CCD diffractometer	1686 independent reflections
Radiation source: fine-focus sealed tube	1444 reflections with *I* > 2σ(*I*)
graphite	*R*_int_ = 0.022
φ and ω scans	θ_max_ = 25.0°, θ_min_ = 1.4°
Absorption correction: multi-scan (*SADABS*; Bruker, 2000)	*h* = −17→17
*T*_min_ = 0.878, *T*_max_ = 0.905	*k* = −6→6
5044 measured reflections	*l* = −13→8

### Refinement

**Table:**

Refinement on *F*^2^	Primary atom site location: structure-invariant direct methods
Least-squares matrix: full	Secondary atom site location: difference Fourier map
*R*[*F*^2^ > 2σ(*F*^2^)] = 0.035	Hydrogen site location: inferred from neighbouring sites
*wR*(*F*^2^) = 0.160	H-atom parameters constrained
*S* = 1.02	*w* = 1/[σ^2^(*F*_o_^2^) + (0.133*P*)^2^] where *P* = (*F*_o_^2^ + 2*F*_c_^2^)/3
1686 reflections	(Δ/σ)_max_ = 0.001
123 parameters	Δρ_max_ = 0.35 e Å^−^^3^
0 restraints	Δρ_min_ = −0.40 e Å^−^^3^

### Special details

**Table:**

Geometry. All e.s.d.'s (except the e.s.d. in the dihedral angle between two l.s. planes) are estimated using the full covariance matrix. The cell e.s.d.'s are taken into account individually in the estimation of e.s.d.'s in distances, angles and torsion angles; correlations between e.s.d.'s in cell parameters are only used when they are defined by crystal symmetry. An approximate (isotropic) treatment of cell e.s.d.'s is used for estimating e.s.d.'s involving l.s. planes.
Refinement. Refinement of *F*^2^ against ALL reflections. The weighted *R*-factor *wR* and goodness of fit *S* are based on *F*^2^, conventional *R*-factors *R* are based on *F*, with *F* set to zero for negative *F*^2^. The threshold expression of *F*^2^ > σ(*F*^2^) is used only for calculating *R*-factors(gt) *etc*. and is not relevant to the choice of reflections for refinement. *R*-factors based on *F*^2^ are statistically about twice as large as those based on *F*, and *R*- factors based on ALL data will be even larger.

### Fractional atomic coordinates and isotropic or equivalent isotropic displacement parameters (Å^2^)

**Table:**

	*x*	*y*	*z*	*U*_iso_*/*U*_eq_	
Cl1	0.11833 (4)	1.10777 (11)	0.35541 (6)	0.0638 (3)	
Cl2	0.09727 (5)	0.41534 (13)	0.65874 (6)	0.0751 (3)	
O1	0.39049 (10)	1.0814 (2)	0.40196 (12)	0.0428 (4)	
O2	0.37013 (9)	0.7923 (2)	0.26477 (12)	0.0414 (4)	
C1	0.12123 (14)	0.7578 (4)	0.50648 (19)	0.0483 (6)	
H1	0.0599	0.7995	0.5178	0.058*	
C2	0.17289 (14)	0.8801 (3)	0.43034 (18)	0.0415 (5)	
C3	0.26501 (12)	0.8219 (3)	0.41278 (16)	0.0361 (5)	
C4	0.30282 (15)	0.6348 (4)	0.47363 (19)	0.0455 (5)	
H4	0.3642	0.5922	0.4629	0.055*	
C5	0.25309 (15)	0.5092 (4)	0.5495 (2)	0.0498 (6)	
H5	0.2801	0.3841	0.5894	0.060*	
C6	0.16219 (16)	0.5739 (4)	0.56487 (19)	0.0469 (6)	
C7	0.32422 (14)	0.9531 (3)	0.33360 (17)	0.0388 (5)	
H7	0.2855	1.0590	0.2857	0.047*	
C8	0.44930 (15)	1.2158 (4)	0.3327 (2)	0.0492 (6)	
H8A	0.4948	1.2992	0.3814	0.059*	
H8B	0.4118	1.3298	0.2898	0.059*	
C9	0.5000	1.0610 (4)	0.2500	0.0365 (6)	
C10	0.42682 (15)	0.9102 (4)	0.18627 (17)	0.0427 (5)	
H10A	0.3877	1.0073	0.1357	0.051*	
H10B	0.4575	0.7965	0.1402	0.051*	

### Atomic displacement parameters (Å^2^)

**Table:**

	*U*^11^	*U*^22^	*U*^33^	*U*^12^	*U*^13^	*U*^23^
Cl1	0.0494 (4)	0.0675 (5)	0.0753 (6)	0.0257 (3)	0.0111 (3)	0.0162 (3)
Cl2	0.0745 (6)	0.0858 (6)	0.0674 (6)	−0.0222 (3)	0.0251 (4)	0.0137 (3)
O1	0.0476 (9)	0.0412 (8)	0.0411 (9)	−0.0055 (6)	0.0148 (7)	−0.0084 (5)
O2	0.0405 (8)	0.0478 (8)	0.0371 (8)	−0.0064 (6)	0.0124 (6)	−0.0095 (6)
C1	0.0344 (10)	0.0593 (13)	0.0524 (14)	0.0033 (9)	0.0125 (9)	−0.0040 (10)
C2	0.0357 (10)	0.0456 (12)	0.0435 (12)	0.0069 (8)	0.0048 (8)	−0.0030 (8)
C3	0.0316 (10)	0.0439 (10)	0.0331 (10)	0.0029 (8)	0.0033 (8)	−0.0044 (8)
C4	0.0355 (11)	0.0532 (13)	0.0480 (13)	0.0095 (8)	0.0043 (9)	0.0047 (9)
C5	0.0486 (12)	0.0515 (12)	0.0491 (13)	0.0040 (10)	0.0016 (10)	0.0098 (10)
C6	0.0476 (13)	0.0525 (12)	0.0414 (12)	−0.0086 (9)	0.0108 (9)	−0.0005 (9)
C7	0.0348 (10)	0.0474 (11)	0.0347 (11)	0.0071 (8)	0.0052 (8)	0.0018 (8)
C8	0.0559 (14)	0.0384 (11)	0.0554 (14)	−0.0039 (9)	0.0225 (11)	−0.0064 (9)
C9	0.0404 (15)	0.0340 (13)	0.0362 (15)	0.000	0.0114 (11)	0.000
C10	0.0417 (11)	0.0554 (12)	0.0315 (11)	−0.0013 (8)	0.0082 (9)	−0.0012 (8)

### Geometric parameters (Å, °)

**Table:**

Cl1—C2	1.737 (2)	C4—H4	0.9300
Cl2—C6	1.741 (2)	C5—C6	1.379 (3)
O1—C7	1.417 (3)	C5—H5	0.9300
O1—C8	1.431 (2)	C7—H7	0.9800
O2—C7	1.414 (2)	C8—C9	1.531 (2)
O2—C10	1.434 (2)	C8—H8A	0.9700
C1—C6	1.373 (3)	C8—H8B	0.9700
C1—C2	1.386 (3)	C9—C10^i^	1.525 (2)
C1—H1	0.9300	C9—C10	1.525 (2)
C2—C3	1.391 (3)	C9—C8^i^	1.531 (2)
C3—C4	1.384 (3)	C10—H10A	0.9700
C3—C7	1.499 (3)	C10—H10B	0.9700
C4—C5	1.378 (3)		
			
C7—O1—C8	110.95 (15)	O1—C7—C3	107.23 (15)
C7—O2—C10	111.11 (15)	O2—C7—H7	110.1
C6—C1—C2	118.81 (19)	O1—C7—H7	110.1
C6—C1—H1	120.6	C3—C7—H7	110.1
C2—C1—H1	120.6	O1—C8—C9	111.43 (16)
C1—C2—C3	121.53 (19)	O1—C8—H8A	109.3
C1—C2—Cl1	117.74 (15)	C9—C8—H8A	109.3
C3—C2—Cl1	120.73 (16)	O1—C8—H8B	109.3
C4—C3—C2	117.28 (19)	C9—C8—H8B	109.3
C4—C3—C7	119.37 (17)	H8A—C8—H8B	108.0
C2—C3—C7	123.34 (17)	C10^i^—C9—C10	110.8 (2)
C5—C4—C3	122.52 (19)	C10^i^—C9—C8^i^	107.54 (12)
C5—C4—H4	118.7	C10—C9—C8^i^	110.94 (12)
C3—C4—H4	118.7	C10^i^—C9—C8	110.94 (12)
C4—C5—C6	118.3 (2)	C10—C9—C8	107.54 (12)
C4—C5—H5	120.9	C8^i^—C9—C8	109.1 (2)
C6—C5—H5	120.9	O2—C10—C9	110.67 (14)
C1—C6—C5	121.6 (2)	O2—C10—H10A	109.5
C1—C6—Cl2	119.24 (17)	C9—C10—H10A	109.5
C5—C6—Cl2	119.17 (18)	O2—C10—H10B	109.5
O2—C7—O1	110.06 (16)	C9—C10—H10B	109.5
O2—C7—C3	109.08 (16)	H10A—C10—H10B	108.1
			
C6—C1—C2—C3	−0.6 (3)	C8—O1—C7—O2	62.5 (2)
C6—C1—C2—Cl1	178.60 (17)	C8—O1—C7—C3	−179.01 (15)
C1—C2—C3—C4	0.7 (3)	C4—C3—C7—O2	47.8 (2)
Cl1—C2—C3—C4	−178.44 (16)	C2—C3—C7—O2	−133.17 (19)
C1—C2—C3—C7	−178.38 (19)	C4—C3—C7—O1	−71.4 (2)
Cl1—C2—C3—C7	2.5 (3)	C2—C3—C7—O1	107.7 (2)
C2—C3—C4—C5	−0.4 (3)	C7—O1—C8—C9	−57.8 (2)
C7—C3—C4—C5	178.70 (19)	O1—C8—C9—C10^i^	−69.6 (2)
C3—C4—C5—C6	0.0 (4)	O1—C8—C9—C10	51.7 (2)
C2—C1—C6—C5	0.1 (3)	O1—C8—C9—C8^i^	172.1 (2)
C2—C1—C6—Cl2	−178.77 (16)	C7—O2—C10—C9	59.2 (2)
C4—C5—C6—C1	0.2 (3)	C10^i^—C9—C10—O2	69.36 (13)
C4—C5—C6—Cl2	179.05 (17)	C8^i^—C9—C10—O2	−171.24 (15)
C10—O2—C7—O1	−63.4 (2)	C8—C9—C10—O2	−52.1 (2)
C10—O2—C7—C3	179.21 (15)		

Symmetry codes: (i) −*x*+1, *y*, −*z*+1/2.

### Hydrogen-bond geometry (Å, °)

**Table:**

*D*—H···*A*	*D*—H	H···*A*	*D*···*A*	*D*—H···*A*
C5—H5···O2^ii^	0.93	2.58	3.425 (3)	152
C7—H7···Cl1	0.98	2.60	3.113 (2)	113

Symmetry codes: (ii) *x*, −*y*+1, *z*+1/2.
